# The evolution of compliance in the human lateral mid-foot

**DOI:** 10.1098/rspb.2013.1818

**Published:** 2013-10-22

**Authors:** Karl T. Bates, David Collins, Russell Savage, Juliet McClymont, Emma Webster, Todd C. Pataky, Kristiaan D'Aout, William I. Sellers, Matthew R. Bennett, Robin H. Crompton

**Affiliations:** 1 Department of Musculoskeletal Biology, Institute of Aging and Chronic Disease, University of Liverpool, Sherrington Buildings, Ashton St., Liverpool, L69 3GE, UK; 2 Department of Bioengineering, Shinshu University, Nagano-ken, Tokida 3-15-1, Ueda-shi 386-8567, Japan; 3 Department of Biology, University of Antwerp, Campus Drie Eiken D.C.1.08, Universiteitsplein 1, 2610 Antwerpen, Belgium; 4 Faculty of Life Sciences, University of Manchester, Michael Smith Building, Oxford Road, Manchester, M13 9PT, UK; 5 School of Applied Sciences, Bournemouth University, Talbot Campus, Fern Barrow, Poole, Dorset BH12 5BB, UK

**Keywords:** foot pressure, mid-foot, locomotion, topological analysis, bipedalism

## Abstract

Fossil evidence for longitudinal arches in the foot is frequently used to constrain the origins of terrestrial bipedality in human ancestors. This approach rests on the prevailing concept that human feet are unique in functioning with a relatively stiff lateral mid-foot, lacking the significant flexion and high plantar pressures present in non-human apes. This paradigm has stood for more than 70 years but has yet to be tested objectively with quantitative data. Herein, we show that plantar pressure records with elevated lateral mid-foot pressures occur frequently in healthy, habitually shod humans, with magnitudes in some individuals approaching absolute maxima across the foot. Furthermore, the same astonishing pressure range is present in bonobos and the orangutan (the most arboreal great ape), yielding overlap with human pressures. Thus, while the mean tendency of habitual mechanics of the mid-foot in healthy humans is indeed consistent with the traditional concept of the lateral mid-foot as a relatively rigid or stabilized structure, it is clear that lateral arch stabilization in humans is not obligate and is often transient. These findings suggest a level of detachment between foot stiffness during gait and osteological structure, hence fossilized bone morphology by itself may only provide a crude indication of mid-foot function in extinct hominins. Evidence for thick plantar tissues in *Ardipithecus ramidus* suggests that a human-like combination of active and passive modulation of foot compliance by soft tissues extends back into an arboreal context, supporting an arboreal origin of hominin bipedalism in compressive orthogrady. We propose that the musculoskeletal conformation of the modern human mid-foot evolved under selection for a functionally tuneable, rather than obligatory stiff structure.

## 1. Introduction

The human foot is considered one of our most distinctive morphological and functional features, yet few of the many hypothesized form–function relationships associated with its mechanics and evolution have ever been tested [1]. Among the most oft-cited remain two early qualitative studies [2,3], which claim that humans possess a mid-foot stabilized in bone that allows the metatarsals to act as an efficient propulsive lever, while by contrast, non-human apes (NHAs) use much greater mid-foot mobility, through a ‘freely movable’ transverse tarsal joint. This results in propulsive forces and peak plantar pressures being exerted under the lateral mid-foot in NHAs, not under the metatarsal heads and phalanges as in humans [2,3].

This apparent dichotomy has provided anthropology with a crucial interpretative paradigm: that fossil evidence for longitudinal arches of the foot and a mid-foot seemingly stabilized in bone can be used to constrain the time of appearance of terrestrial bipedality and modern foot function in human ancestors [2–5]. However, the nature and magnitude of functional differences among the feet of living apes currently remains poorly constrained by quantitative data, and our ability to diagnose foot and limb mechanics in the hominin lineage is largely dependent on a highly qualitative view of the modern human foot.

The lack of quantitative data in part owes to inherent difficulties in quantifying mid-foot mechanics *in vivo*. Recently, invasive ‘bone pin’ approaches [6] have shown extensive variation in joint excursions between human subjects, but such studies have so far been restricted to very small sample sizes. Practical difficulties are amplified in studies of NHA feet, and subsequently no quantitative data currently exist on their mid-foot kinematics during terrestrial locomotion. The difficulty of directly assessing internal motion and forces in the primate foot means that external measures of foot mechanics, in the form of plantar pressure records, have become crucial to our understanding of foot function during locomotion [5,7–11].

The advent of pressure-recording treadmills enables us to greatly increase sample sizes for foot pressure and test ideas about human foot function more robustly. Herein, we analyse a unique dataset of over 21 500 human plantar pressure records collected at a standardized walking speed using a Zebris FDM-T treadmill. Analysis of this new dataset in comparison to pressure records of two NHAs (bonobos and orangutans) allows us to begin to quantitatively constrain the nature and magnitude of functional differences between the feet of human and NHAs, and shed light on the evolution of compliance in the hominin foot.

## 2. Material and methods

### (a) Data collection

All human pressure data were collected on a Zebris FDM-THM foot pressure-sensing treadmill. A total of 45 human subjects (22 males and 23 females, aged 18–68 years) without any limb abnormalities or injuries walked barefoot at a constant speed of 1.1 m s^−1^ for 5 min. Subjects varied in cardiovascular/respiratory fitness, and 1.1 m s^−1^ was chosen because experimentation revealed it to be a comfortable pace for 5 min continuous walking in all subjects. Preliminary analysis from a subset of subjects at 1.1–1.5 m s^−1^ reveals no statistically significant differences in peak plantar pressure patterns (see electronic supplementary material, figure S1), hence we are confident that our results are not strongly influenced by the choice of walking speed. Kinematic data were collected synchronously using an integrated 12-camera Qualisys motion capture system at a frequency of 100 Hz, with an array of 29 spherical markers across the body.

Foot pressure in voluntary, unelicited bipedalism of bonobos was recorded in a large enclosure at Planckendael Zoo, at a resolution of 16 pixels cm^−2^, using RSscan Footscan 0.6 m pressure plates set in a wooden walkway. Records were also made of a juvenile orangutan at Twycross Zoo walking over a 1 m Footscan plate with the same resolution, placed on a rigid floor. The orangutan was guided by its habitual keeper using a light hand touch, with the keeper being requested to provide no support. Only records where no such support was evident were retained for analysis. All such work received prior approval of zoo authorities and adhered strictly to the Association for the Study of Animal Behaviour and Animal Behaviour Society (ASAB/ABS) Guidelines.

### (b) Data processing and analysis

Pressure data from the treadmill corresponding to the maximum pressure recorded in each pressure cell during each subject's footfalls were extracted using a custom-written C program, yielding 432–562 records per subject. Pedobarographic images were then registered using an algorithm that minimized the mean squared error between the images such that homologous structures optimally overlap [9,11]. All image processing and analysis were conducted using MATLAB (MathWorks, USA).

To examine variability in mid-foot pressure, we subjectively defined the mid-foot region in each subject's left and right mean record (see electronic supplementary material, figures S2 and S3). We could not derive an objective quantitative way to define the mid-foot region that was repeatable across all subjects owing to significant variation in pressure distributions and foot proportions (detail in the electronic supplementary material, S1). Based on the mid-foot definition in mean prints, left and right prints were then sorted into subsampled groups in two different ways; first, based on a clinical threshold [7] for investigating possible mid-foot collapse (i.e. (i) mid-foot peak pressure less than 200 kPa or (ii) mid-foot peak pressure greater than 200 kPa) and second, based on the mid-foot peak pressure as a percentage of overall peak pressure (see electronic supplementary material, figure S4). In addition to analysing the relative frequency within these categories, we conducted topological statistical comparisons [9,11] of these subsampled groups to identify any systematic changes in pressure distribution that correlate with differences in mid-foot pressure.

## 3. Results

### (a) Human and non-human ape foot pressures

**Figure 1.**
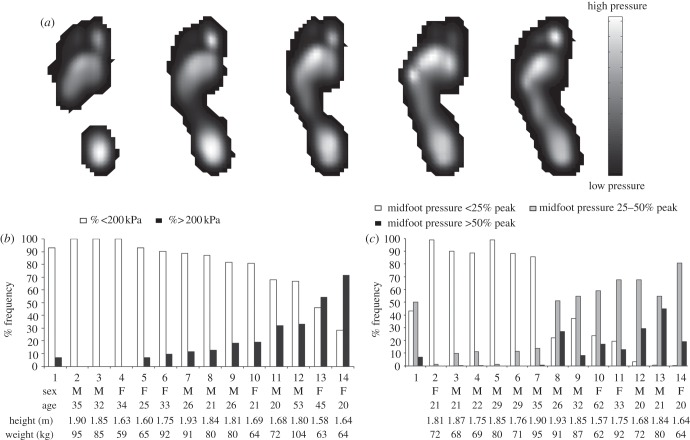
(*a*) Example mean peak plantar pressure records illustrating the range of inter-subject variation in mid-foot pressure. (*b*) Frequency plot showing the percentage of footfalls above and below a 200 kPa threshold [7] for (1) the mean of the entire human dataset and (2–14) a selection of subjects. (*c*) Plot showing the frequency distribution of footfall categories based on mid-foot pressure as a percentage of overall peak pressure individual footfalls for (1) the mean of the entire human dataset and (2–14) a selection of subjects.

Our analysis demonstrates that modern Western human plantar pressures vary considerably among and within individuals (figure 1). Records with elevated lateral mid-foot pressure occur quite frequently (figure 1*b*,*c*). In clinical practice, mid-foot peak pressure of 200 kPa or more is regarded as meriting investigation for risk of mid-foot collapse [7]. In our dataset, 30 out of 45 healthy individuals (67%) recorded at least one footfall in both feet where the lateral mid-foot exceeded this criterion during just 5 min of steady-state walking (figure 1*b*). In total, approximately 7% of footsteps recorded exhibited mid-foot pressure greater than 200 kPa, but some individuals produced such records more frequently: in four subjects more than 15% and in two subjects more than 55%, of footsteps (figure 1*b*).

We also categorized individual prints based on mid-foot peak pressure as a percentage of peak pressure in each footfall, which permits comparison to uncalibrated records for non-human primates. This analysis further emphasizes the magnitude of intra- and inter-subject variation in relative mid-foot pressure in modern Western humans. The dataset contains individuals who exert little or no lateral mid-foot pressure (2–7 in figure 1*c*), individuals with relatively even distribution across low to high mid-foot pressure categories (8–11 in figure 1*c*) and subjects that consistently exert relatively high pressures under the lateral mid-foot (12–14 in figure 1*c*). The presence or absence of footfalls with absolutely (figure 1*b*) or relatively high pressure (figure 1*c*) does not show any systematic relationship with gender, age, height or weight (figure 1).

**Figure 2.**
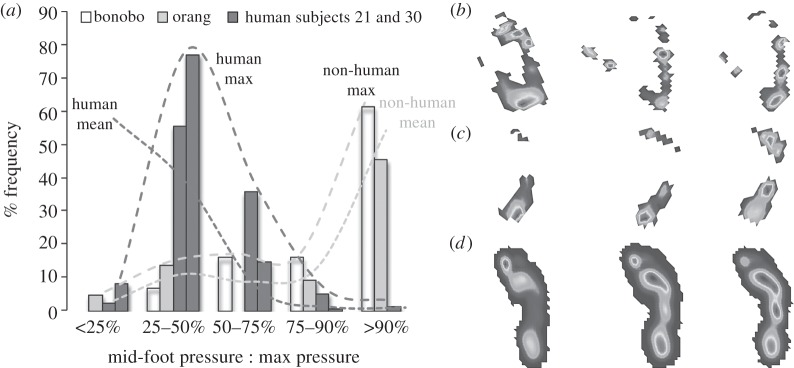
Comparison of relative mid-foot pressures in human and NHAs. (*a*) Plot showing the frequency distribution of footfalls categories based on mid-foot pressure as a percentage of overall peak pressure individual footfalls for the bonobo, orangutan and two human subjects (numbers 21 and 30 in electronic supplementary material 2) with relatively frequent occurrences of footfalls with high mid-foot pressures. Maximum lines represent the human and non-human subjects with the most extreme right skew to their mid-foot pressure distributions, whereas the mean lines represent the average frequency for each mid-foot pressure category in the full human and non-human datasets. (*b*–*d*) Mean plantar pressure records for (*b*) bonobos, (*c*) orangutans and (*d*) human subject 35 for the mid-foot pressure categories shown in (*a*) (left, mid-foot pressure less than 25% peak pressure; middle, mid-foot pressure 50–75% peak pressure; right, mid-foot pressure greater than 90% pressure peak).

Repeating this analysis on a small number of pressure records from bipedally walking bonobos (*N* = 11) and orangutans (*N* = 8) reveals an equally striking range of relative mid-foot pressure (figure 2). As in habitually shod Western humans, bonobos and orangutans (the most arboreal of the great apes [12]) produced records in which mid-foot pressure ranged from less than 25% to in excess of 90% peak plantar pressure (figure 2). While the central tendency of human mid-foot pressure clearly differs from the mean mid-foot pressure in the bonobo and orangutan, mid-foot compliance in living apes, including humans, seemingly represents a functional continuum between the relatively stiff feet of humans and the more compliant feet of NHAs (figure 2), with clear and previously unquantified overlap in ranges of variation.

### (b) Human lateral mid-foot kinematics

**Figure 3.**
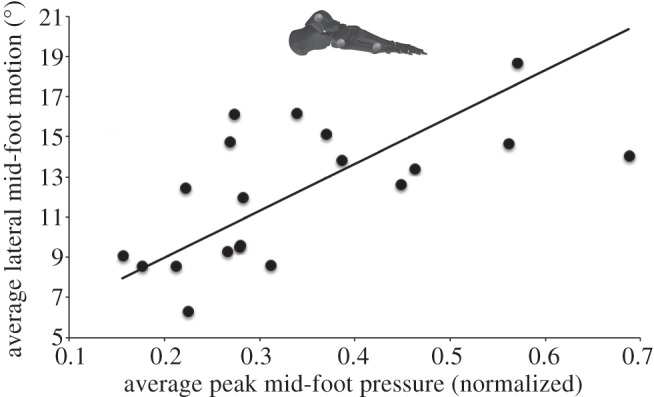
The relationship between average stance phase lateral mid-foot motion in the sagittal plane and the average peak mid-foot pressure in 20 human subjects. The moderate positive linear relationship indicates that subjects that habitually exhibit greater lateral mid-foot motion also on average produce higher peak pressures under the lateral mid-foot. Average lateral mid-foot motion was measured as the total stance phase angular excursion between markers on the lateral ankle, proximal and distal metatarsal five.

Lateral mid-foot kinematics was quantified during treadmill walking using skin markers on the lateral ankle, and proximal and distal heads of the fifth metatarsal (figure 3). Average angular excursions varied considerably between subjects, ranging from 6.3° to 18.7° (figure 3). Reduced major axis regression (23.306*x* + 4.3225; *r*^2^ = 0.61698) shows that a moderate positive linear relationship exists between average lateral mid-foot peak pressure and habitual lateral mid-foot motion, indicating that subjects that habitually exhibit greater lateral mid-foot motion do also on average produce higher peak pressures under the lateral mid-foot (figure 3).

## 4. Discussion

### (a) Habitual compliance in the hominin lateral mid-foot

Our unique dataset demonstrates that lateral arch stabilization in humans is not obligate and is often transient (figures 1–3). Thus, while the mean tendency of habitual mechanics of the mid-foot in healthy humans is consistent with the traditional concept of the lateral mid-foot as a relatively rigid or stabilized structure compared to that of other apes, the dataset is also equally characterized by a range of variation that includes individuals with high step-to-step variability in arch compliance and individuals that exhibit consistently high pressure under the lateral mid-foot. Indeed, it would seem that bipedalism in great apes generally is characterized by high intra-subject (i.e. step-to-step) variation in mid-foot pressures. This variation results in overlap in relative mid-foot pressure between great ape species, indicating that the long-standing qualitative dichotomy in external mid-foot function during terrestrial walking proposed by Elftman & Manter [2,3] between humans and other great apes does not strictly exist.

That overlap in relative mid-foot pressures occurs is perhaps even more surprising given the demography of the subjects. All were habitually shod, healthy individuals, with only seven over the age of 30. There have been isolated reports of qualitatively defined ‘mid-tarsal breaks’ in pressure records and footprints made by habitually unshod individuals [8,9], and a recent study showed that Western subjects differed strongly from habitually shod and unshod Indian populations in having higher, less diffuse peak pressures under the heel, metatarsals and hallux [10].

That subjects exhibiting greater mobility show a clear tendency to higher peak pressures under the lateral mid-foot provides support for a causative link between kinematics and pressure (figure 3). The statistical relationship between habitual lateral mid-foot motion and average lateral mid-foot peak pressures (figure 3) may be considered strong given that the human foot represents a biological system with 26 moving parts. That the relationship is not stronger probably indicates that other factors (e.g. inter-subject morphological variation) are also likely to contribute relative pressure patterns between subjects. However, some caution is warranted in interpreting this data. All kinematic analyses based on surface markers undoubtedly suffer from skin–motion artefacts, which may impact on the accuracy with which measured angular changes reflect relative bone motion. For example, it is possible that some overall medio-lateral rolling of foot may be contributing to observed patterns (figure 3). Nevertheless, these results require us to re-evaluate our understanding of stiffness and mobility in the lateral mid-foot of humans (and great apes generally) during locomotion.

Reliable mid-foot kinematic data for NHAs are currently unavailable so we cannot assess whether the overlap in relative mid-foot pressure is accompanied by overlap in joint motions. Irrespective of whether overlap in kinematics exists, the mechanisms underpinning control of foot compliance in human and NHAs almost certainly differ in some respects. For example, all living NHAs lack the plantar aponeurosis (PA) and have a much higher muscle : tendon mass ratio than humans (e.g. all but gibbons lack a substantial Achilles tendon [8]). This particular distinction is consistent with humans having experienced some degree of selective pressure for effective cursorial (and hence terrestrial) locomotion [8].

However, although more inclined to use the ground than Asian apes, African apes vary considerably in their degree of arboreality: lowland gorillas are regularly arboreal and females predominantly so [13], mountain gorillas and common chimpanzees predominantly terrestrial but bonobos intermediate [14]. While arboreal activities such as hallucal grasping might be expected to be reflected in osteological differences between human and NHA feet, caution must be exercised here too: it is the most arboreal African ape, the lowland gorilla, whose foot most resembles that of humans in bony proportions [15] and biomechanical function [16]. Understanding the mechanical and neuromuscular control of foot compliance used during both arboreal and terrestrial locomotion in living apes (versus maximal permissible joint motion) is thus crucial to our understanding of foot evolution and the origins of bipedality.

### (b) The evolution of the longitudinal arches of the foot

Direct tests of morpho-functional hypotheses using experimental data are often challenging as the inherent complexity of biological systems makes isolating the specific effect of the structure or function of interest difficult, if not impossible. However, step-to-step variation in mid-foot pressure within individuals provides a case in which morphology is inherently constant, allowing the effects of functional variation (i.e. mid-foot compliance) to be objectively quantified.

**Figure 4.**
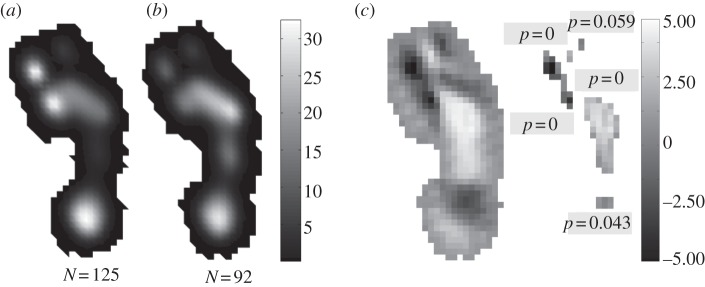
Topological statistical comparisons of peak plantar pressure records with a mid-foot pressure less than 25% peak pressure against those in which mid-foot pressure is greater than 50% overall peak pressure in a single human subject. (*a*) Mean peak plantar pressure for records with mid-foot pressures less than 25% peak pressure. (*b*) Mean peak plantar pressure for records with mid-foot pressures greater than 50% peak pressure. (*c*) Statistical parametric maps (SPM) showing areas of difference and levels of statistical significance between the means and their populations. In SPMs, lighter shades indicate areas of higher pressure in the ‘mid-foot pressure greater than 50% peak pressure’ category, whereas darker areas indicate relatively higher pressures in the ‘mid-foot pressure less than 25% peak pressure’ category.

Figure 4 shows topological statistical comparisons, in a single human subject, of plantar pressure records with lateral mid-foot pressure less than 25% peak pressure against those in which mid-foot pressure is greater than 50% overall peak pressure. These indicate that pressure under the anterior heel, and particularly the first metatarsal head and hallux, is significantly lower when mid-foot pressure is high. Remarkably, this same pattern is found in all subjects that produced plantar pressures in which mid-foot pressure exceeded 50% overall peak pressure (see electronic supplementary material, figure S5), providing strong evidence of a ubiquitous functional coupling mechanism in the human foot, in which lateral mid-foot compliance is inversely related to the pressure exerted by the medial metatarsal heads and hallux. To our knowledge, this is the first direct experimental evidence that arch stiffness in isolation increases medio-lateral force transfer and medial forefoot propulsion in human walking.

It was proposed some time ago [17] that the human foot becomes stiffer in late stance as the PA is stretched around the metatarsal heads by the dorsiflexing phalanges, and recently a dynamic foot model demonstrated that muscular loading generates tension in the PA even before heel strike [18]. Preloading the PA not only prevents arch collapse but also leads to increased PA tension in late stance, which increases the rearward pull on the forefoot, and thus assists propulsion [12,18]. We speculate that the coupling between mid-foot and forefoot pressure that we have identified (figure 4), reflects variation in loading of the PA, and subsequently that PA-controlled arch stiffness represents an important step-to-step stability mechanism in human walking. Widening this analysis to include whole-body motion and lower limb muscle activation data might provide insights into the role of such variations in foot mechanics (pressures and kinematics) in the stability and control of human walking.

Resistance to flexion in the human lateral mid-foot has traditionally been attributed to osteological ‘locking’ of the transverse tarsal joint [2,3]. Although plantar ligaments are acknowledged to contribute to mid-foot stiffness [5], it is generally held that ‘locking’ is primarily in bone, served by a large cuboid ‘peg’ that slots into a corresponding groove on the plantar calcaneus, a feature supposedly absent in non-human great apes [2]. This remains an as-yet-untested proposition and our results (figures 1–4) cast significant doubt on its validity, at the very least as a ubiquitous functional constraint within modern humans. The foot is clearly a highly complex and integrated system, with various mechanisms for modulating activity during locomotion. The intrinsic functional coupling between mid- and forefoot indicated by within-subject pressure patterns (figure 4; also the electronic supplementary material, figure S5) strongly suggests that soft tissues provide the primary control on the variations in mid-foot mobility or compliance observed in our analysis.

The clear functional relationship between mid-foot compliance and pressures under the forefoot (figure 4; also the electronic supplementary material, figure S5) requires refinement of the long-standing hypothesis concerning the evolution of the longitudinal arches of the modern human foot. Increased medial forefoot pressures in footfalls with low mid-foot pressures (figure 4) provide direct experimental support for a link between mid-foot stiffness and medial forefoot propulsion, as traditionally proposed. However, our results strongly suggest that the modern human lateral mid-foot is more compliant during habitual locomotion than previously hypothesized (figures 1–4; see also [5,6]), leading us to speculate that the conformation of bones, muscles and passive tissues making up the longitudinal arches evolved to allow active and passive modulation of mid-foot compliance during locomotion as a mechanism for aiding stability during foot–substrate interaction. Thus, rather than as structures permanently stiffened by osteological constraints, we suggest that the longitudinal arches of the human foot are better considered as functionally tuneable structures, albeit with more limited maximal compliance than NHAs.

### (c) The origins of hominin bipedality and ‘modern’ human foot function

The high levels of step-to-step variation in compliance of the lateral arch, and an overlap in relative lateral mid-foot pressures between humans and other great apes (figure 2) described herein potentially impact upon functional interpretations of fossil foot bones and on ideas surrounding the temporal and ecological origins of hominin bipedality. Without further knowledge of the relative contributions of osteology, muscle and other passive soft tissues to mid-foot support during terrestrial locomotion in living taxa, the presence of longitudinal arches in fossil foot bones may be a relatively poor indication of where individual extinct hominin species might plot on the locomotor continuum formed by hominins, panins, gorillines and pongines (figure 2).

That bone morphology fails to unambiguously predict foot function in a terrestrial context perhaps explains why little or no consensus exists about the presence of a medial longitudinal arch and/or a stabilized lateral mid-foot in all fossil hominins that predate *Homo erectus*; for example, the contrasting interpretations of the available *Australopithecus afarensis* foot bones from AL-288–1 and AL-333 [19–21]. Conflicting functional signals in osteological remains, and further discoveries suggesting a mosaic of supposed ‘arboreal’ and ‘terrestrial’ adaptations in the foot of early (StW 573 [22]) and late (*Australopithecus sediba* [23]) australopiths, and most recently in the Woranso–Mille hominin BRTVP-2/73 [24] have led some to propose multiple paths to terrestrial bipedality [19] and others a secondary increase in arboreality within lineages [24]. More parsimoniously, perhaps, the overlap demonstrated here between mid-foot plantar pressures in human and NHAs might suggest that the articular complexity of the 26 bones and 80+ ligaments of the foot results in a high degree of functional redundancy so that similar external biomechanics can be generated by different musculoskeletal conformations.

*Ardipithecus ramidus* (4.4 Ma) currently provides our best insight into the last common ancestor of hominins and panins [25]. Lovejoy *et al*. [25] observed a mosaic of terrestrial and arboreal features in the foot of *Ar. ramidus*; high robusticity of metatarsals II and III might reflect relatively high accelerative forces during forefoot propulsion during terrestrial bouts of locomotion, while a high degree of abduction of the hallux might suggest adaptation to grasping branches [25]. Most relevantly perhaps, Lovejoy *et al*. [25] make an important distinction between bony longitudinal arches and foot stiffness (one clearly supported by our analysis, figures 1–4), noting that retention of thick fibrous plantar tissue probably gave *Ar. ramidus* a stiffer foot than living NHAs, despite the absence of a medial longitudinal arch. Indeed, it is possible that *Ar. ramidus* used a combination of active and passive means for modulating foot compliance step-to-step that is broadly analogous to the PA-based mechanism suggested for humans [11,18], and fundamentally different from the muscle-driven control underpinning the variation we have identified in living NHAs, which lack substantial passive plantar tissues (figure 2). Thus our demonstration of overlapping external foot function in living apes is fully consonant with the hypothesis of a mixed arboreal–terrestrial ecology in *Ar. ramidus*. Retention of primitive features in the hand suggests an absence of suspensory and vertical-climbing specialization [25]), and hence implies origin of hominin bipedality in (arboreal) compressive orthogrady [8,12].

## 5. Conclusion

This study provides the first quantitative analysis of relative mid-foot function in human and non-human great apes during bipedal terrestrial locomotion. It demonstrates for the first time that bipedalism in great apes is characterized by large inter- and intra-individual variation in mid-foot plantar pressure and by inference in joint motion, during stance (figures 1–3). Mid-foot compliance in living apes represents a functional continuum, with clear quantitative overlap between the relatively stiff feet of humans and the more compliant feet of NHAs (figure 2). Systematic and seemingly ubiquitous differences in pressure distribution in humans related to arch compliance provide direct evidence for a link between mid-foot stiffness, medial-to-lateral force transfer and forefoot propulsion (figure 4). Coupling between the mid- and forefoot likely results from variation in muscular preloading of intrinsic soft tissues (particularly the PA), and suggests that *soft* tissues primarily determine the degree of mid-foot compliance/mobility realized during habitual locomotion. We argue that a better understanding of the relative contributions of muscle, bone and passive soft tissues to mid-foot mobility in living taxa is required to make robust inferences about foot function in extinct hominins.

## Notes

All procedures were approved by the University of Liverpool Research Ethics Committee (RETH 0000888).
